# Editorial: Evolution in Neurogenomics

**DOI:** 10.3389/fgene.2023.1220750

**Published:** 2023-06-02

**Authors:** Jiuyong Xie, Robert Friedman

**Affiliations:** ^1^Department of Physiology and Pathophysiology, Max Rady College of Medicine, Rady Faculty of Health Sciences, University of Manitoba, Winnipeg, MB, Canada; ^2^Department of Biological Sciences (Retired), University of South Carolina, Columbia, SC, United States

**Keywords:** neurological disorders, genome, transcriptome, diagnosis, prognosis, machine learning

To encourage further study of neurogenomics and disease by large scale DNA sequencing methods, we have selected five articles in our Research Topic entitled Evolution in Neurogenomics. This Research Topic includes neurodevelopmental disorders and disease, and approaches at the genomic, epigenomic, transcriptomic and epi-transcriptomic levels. In particular, Kim et al. explored clinical phenotypes and genetic variants by whole exome sequencing, a “massively parallel DNA-sequencing” method (Rabbani et al., 2014), in a cohort of pediatric patients with a diverse array of movement disorders. These are disorders that defy strict classification by conventional methodology. They successfully showed the potential of whole exome sequencing as a genetic diagnostic tool for yet another complex neurological disorder (Retterer et al., 2016).

Our second selection of the Research Topic is Akter et al. who surveyed the clinically relevant “copy number variants” across a large number of patients, an underrepresented population in Bangladesh, with neurodevelopmental disorders. They sampled these variants by chromosomal microarray analysis and droplet digital polymerase chain reaction, an approach that is relatively less precise as a diagnostic tool, yet it showed applicability for clinical use and at a relatively low cost. For a more definable pathology, Hu et al. applied a meta-analytical method, including a large number of studies and an overall sample of over 18,000 individuals of Chinese ancestry, and identified three single nucleotide polymorphisms that show association with risk of ischemic stroke.

The next two studies focused on glioma. For the first of these studies, Zhang et al. constructed and validated a risk score model for prognosis of low-grade gliomas. They based their analysis on 14 genes that are chromatin regulators, and included data from single-cell RNA-seq along with clinical data from The Cancer Genome Atlas. For the next study, Zhang et al. reported 12 m6A regulatory genes as putative biomarkers for the prognosis of glioma, a finding that included 1,600 samples. Both are disease association studies that include different methodologies, while the challenge is in synthesizing their findings for advancement of knowledge in neurology and biomedicine.

Likewise, current research in neurogenomics is showing a great potential for association of genetic features with disease, including by genome- and transcriptome-based analyses of genetic variation, long-read RNA sequencing (Gao et al., 2023), and spatial/temporal *in situ* genome and transcriptome mapping (Longo et al., 2021; Payne et al., 2021). The studies of our Research Topic are exemplars of a heterogeneity of techniques for surveying genetic variation, such as by the use of microarray analysis or whole exome sequencing. Of special interest are the DNA sequencing methods that generate very large samples of data, such as in whole exome sequencing. These techniques are both efficient and applicable for biological research since DNA sequencing is trending towards no cost (Wang et al., 2015), while the traditional cost of software-based analysis and human labor is becoming replaceable by advanced machine learning methods (Yang et al., 2020; Bansal et al., 2022).

Furthermore, models such as the Galactica large language model (Taylor et al., 2022) can facilitate the conversion of human readable data and tables to a machine-readable format, and allow for automated construction of biological databases, especially powerful in the case of organization of large samples of data from current DNA sequencing methods. The Galactica model also has a capability for associating biological data, whether processed or not, with scientific knowledge as composed in the common natural languages (Chen, 2022; Taylor et al., 2022). This can lead to a pooling of knowledge across biomedical journals and facilitates a broad and systematic survey of the literature. Moreover, it has a promise for automation of the process of scientific communication and that of data from large scale DNA sequencing (Whang et al., 2023).

The hope is that our Research Topic will stimulate neurogenomic studies that accurately map the genome to pathologies of the brain and the related tissues of the nervous system, while fusing the more traditional approaches with recent techniques of machine learning, as in the sequence-based deep learning models (Vaswani et al., 2017; Poplin et al., 2018; Baid et al., 2023; Liao et al., 2023). These recent techniques extend beyond the practice of data organization and systematic collection of knowledge, but also to application in algorithmic discovery (Fawzi et al., 2022), leading to the potential for automation of machine learning method selection—another area of common interest and applicability to disease association studies. Our Research Topic is composed of studies that are particularly adapted to these approaches for automation of scientific research, leading to an acceleration of scientific discovery. This prediction is not only limited to that of biomedical research, but it is also applicable to the vast collections of data in clinical medicine that is not yet centralized nor fully accessible for machine-based retrieval methods (Topol, 2019).
